# Supplementary material for: Chemical and Biological Aspects of Different Species of the Genus Clinanthus Herb. (Amaryllidaceae) from South America
Source: Molecules. 2023 Jul 14;28(14):5408. doi: 10.3390/molecules28145408 (PMC10385320; doi:10.3390/molecules28145408)
Supplement: Supplementary file 1 [file molecules-28-05408-s001.zip › molecules-2464573-supplementary.pdf]

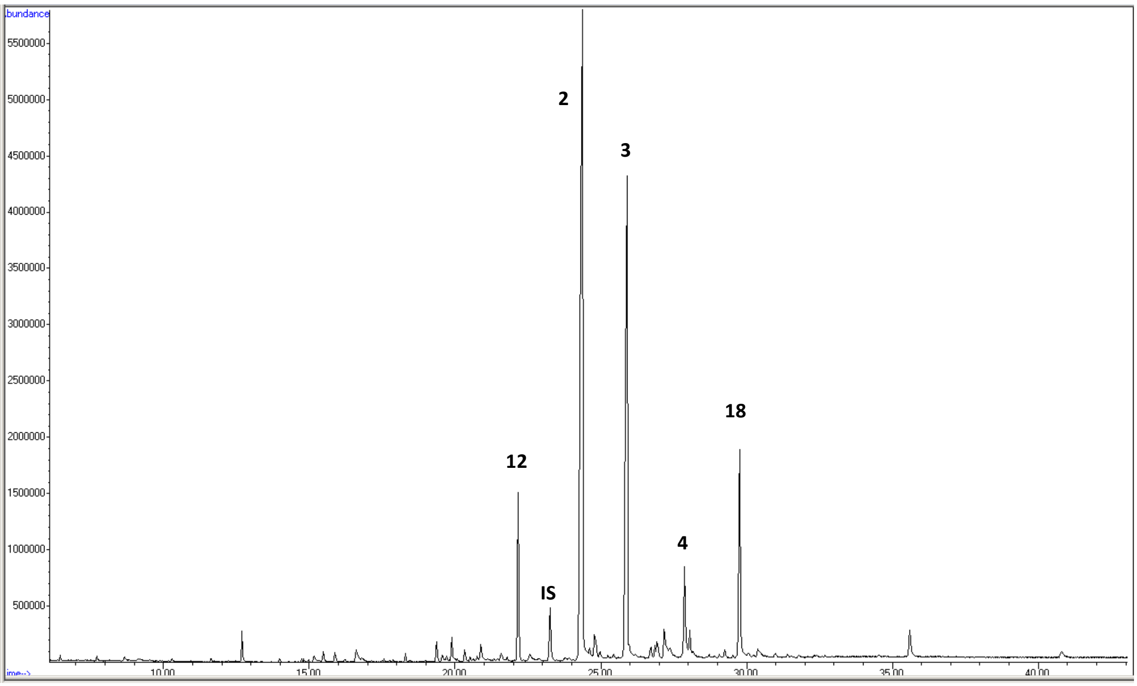

Figure S1. GC chromatogram of the alkaloid extract of *Clinanthus* sp. (sample A).

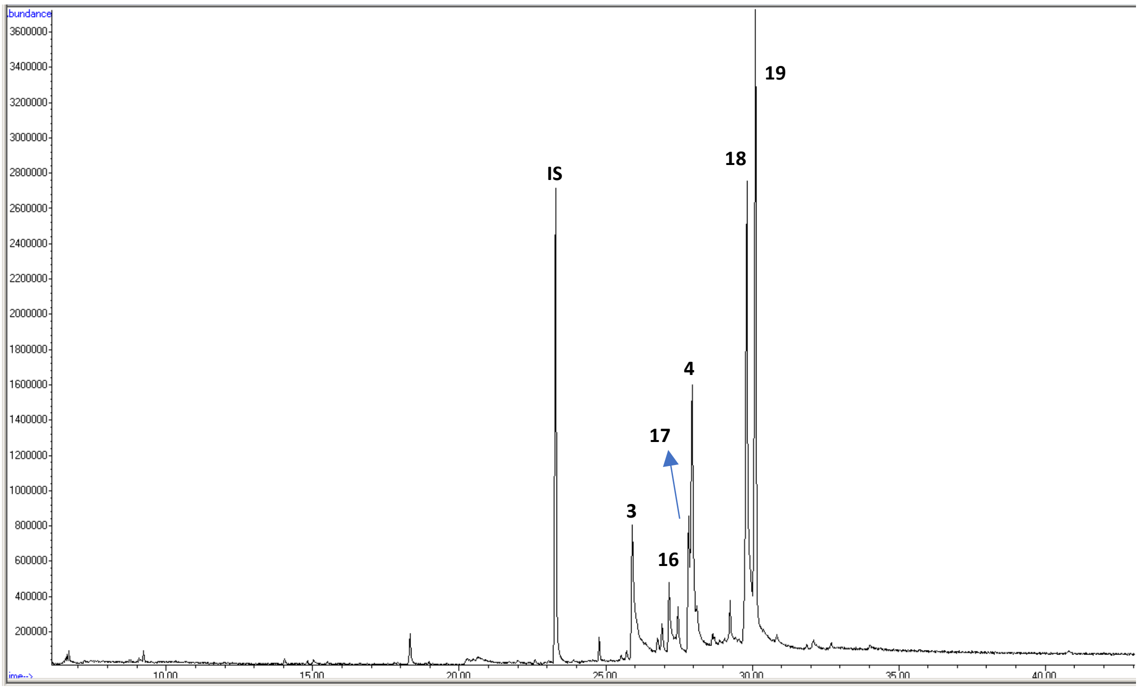

Figure S2. GC chromatogram of the alkaloid extract of *Clinanthus incarnatus* (sample B).

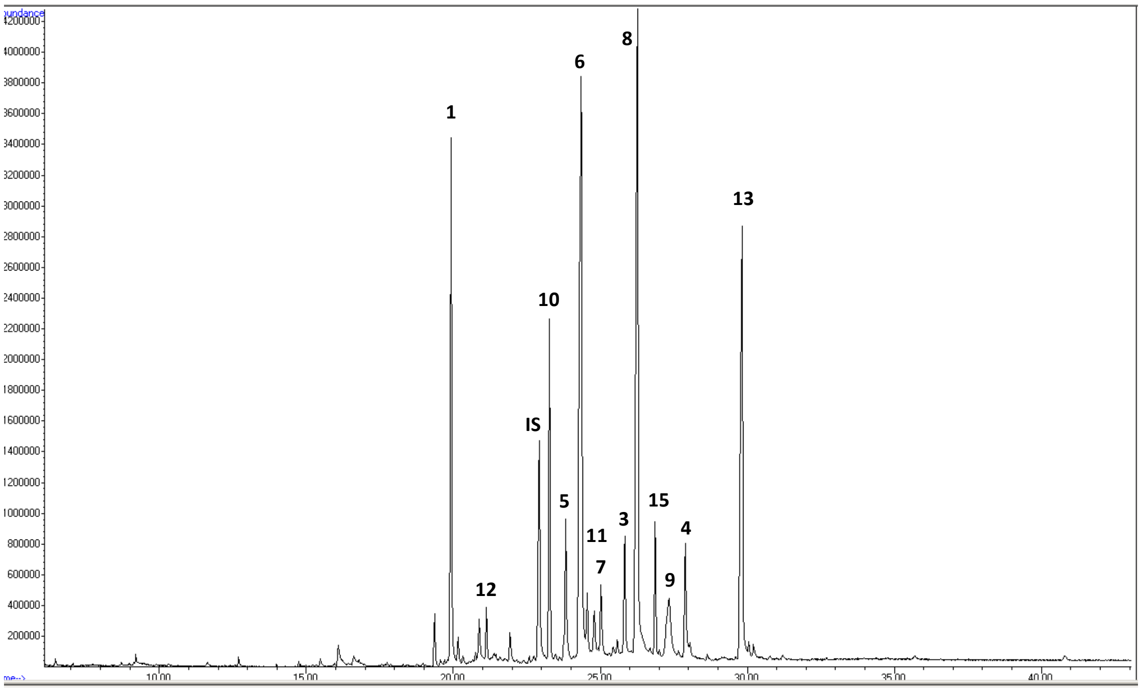

Figure S3. GC chromatogram of the alkaloid extract of *Clinanthus variegatus* from Bolivia (sample C).

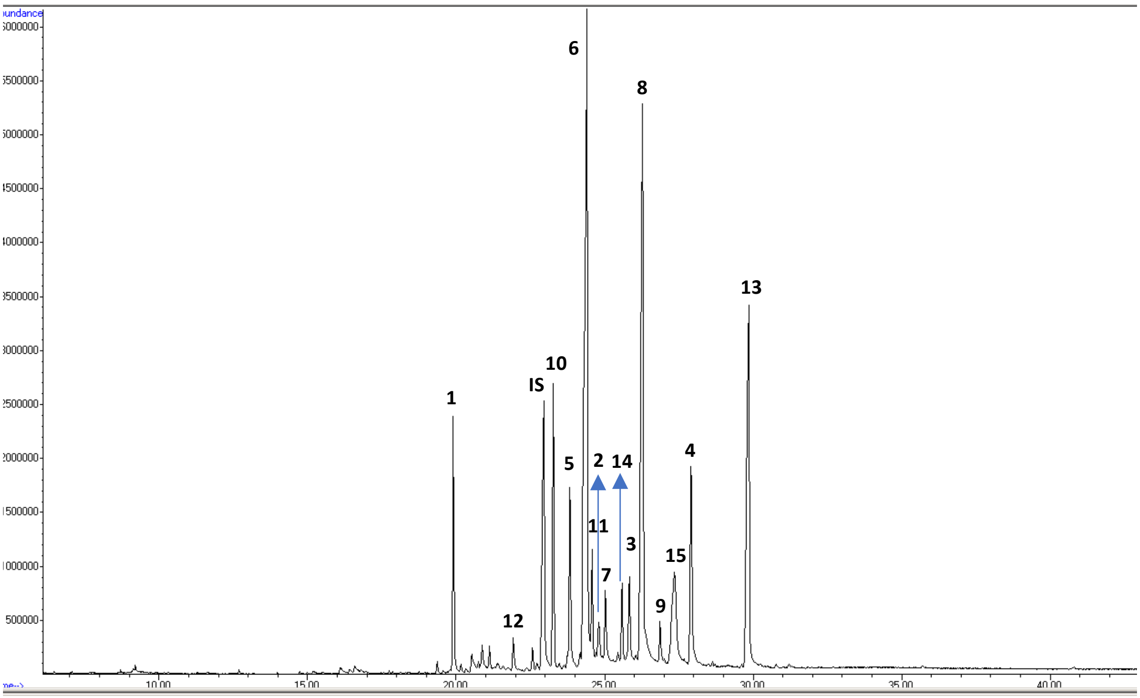

Figure S4. GC chromatogram of the alkaloid extract of *Clinanthus variegatus* from Peru (sample D).
